# A pilot, two-center, sequential dose escalation safety study of alteplase with fresh frozen plasma during normothermic liver perfusion

**DOI:** 10.1097/LVT.0000000000000814

**Published:** 2026-02-24

**Authors:** Christopher J.E. Watson, Stephen Macdonald, Danielle White, Christopher Bridgeman, Rohit Gaurav, Lisa Swift, Rachel Webster, Satheesh Iype, Keziah Crick, Joerg-Matthias Pollok, Carlo D.L. Ceresa, Subhankar Paul, Jack Martin, Sara S. Upponi, Ian Currie, Theodora Foukaneli, Vasilis Kosmoliaptsis, Andrew J. Butler

**Affiliations:** 1 The Roy Calne Transplant Unit, Cambridge University Hospitals NHS Foundation Trust, Hills Road, Cambridge, UK; 2 Department of Surgery, University of Cambridge, Addenbrooke’s Hospital, Cambridge, UK; 3 The National Institute for Health and Care Research (NIHR) Blood and Transplant Research Unit in Organ Donation and Transplantation (NIHR203332), UK; 4 The NIHR Cambridge Biomedical Research Centre (NIHR203312), Cambridge, UK; 5 Specialist Haemostasis Laboratory, Cambridge University Hospitals National Health Service Trust, Cambridge, UK; 6 Department of Hepato-Pancreato-Biliary and Liver Transplant Surgery, Royal Free London NHS Foundation Trust, London; and the Division of Surgery and Interventional Science, University College London, London, UK; 7 Department of Radiology, Cambridge University Hospitals National Health Service Trust, Cambridge, UK; 8 Scottish Liver Transplant Unit, Royal Infirmary, Edinburgh, UK; 9 Department of Haematology, Cambridge University Hospitals National Health Service Trust, Cambridge, UK

**Keywords:** bile duct diseases, fibrin, liver transplantation, perfusion, thrombolytic therapy, tissue plasminogen activator

## Abstract

Donor livers may contain occult fibrin, which is associated with adverse transplant outcomes in livers donated after brain death (DBD) and circulatory death (DCD). Ex situ normothermic machine perfusion (NMP) may allow fibrinolytic therapy before transplantation. Before undertaking a large efficacy study for the prophylaxis of cholangiopathy, we wished to assess the safety of a protocol comprising alteplase and fresh frozen plasma (FFP), the latter as a source of plasminogen, and also to ascertain which of the 4 possible treatment strategies was associated with the greatest fibrin breakdown, as judged by D-dimer release. Eighty livers, 40 DBD and 40 DCD, were randomized to 1 of 5 groups of 16 each, receiving 10 mg or 20 mg alteplase, 1 or 2 units (250 mL) of FFP, with alteplase and FFP being delivered into either the hepatic artery cannula or portal reservoir at the start of NMP. The primary endpoint was bleeding post-transplant. Forty-four of 64 treated livers were transplanted, as were 12 of the 16 control livers receiving FFP alone. There was no increase in post-implant bleeding or blood transfusion requirement of any alteplase-treated liver compared with the FFP alone control group. All 4 alteplase groups were associated with more D-dimer release than the FFP alone control group; 10 mg alteplase was as effective as 20 mg, and delivery into the portal reservoir was as effective as delivery into the hepatic artery cannula. The protocol achieving release of most D-dimers involved 10 mg alteplase being delivered directly into the portal reservoir containing 2 units of FFP. Portal delivery was found to be more straightforward than infusion into the hepatic artery cannula. The combination of alteplase with FFP appeared safe, with no bleeding complications.

## Graphical abstract

**Figure:**
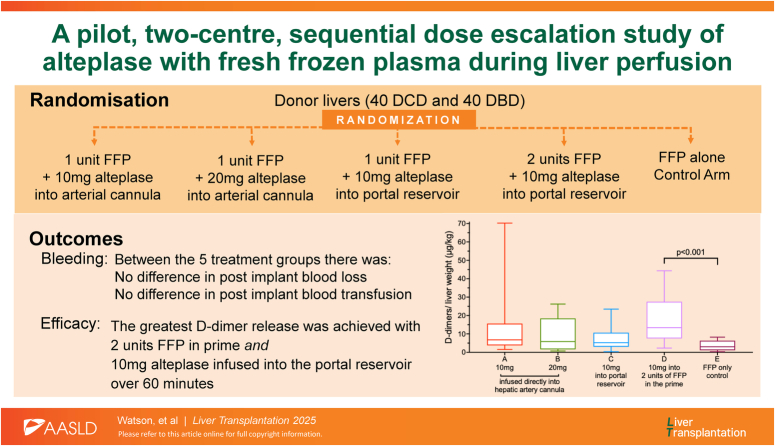

## INTRODUCTION

Donation after circulatory death (DCD), donor age, cold ischemia, and warm ischemia are recognized risk factors for post-transplant cholangiopathy (PTC).^1^ It has also been suggested that fibrin microthrombi, compounded by periods of hypoperfusion, cause stromal infarcts in bile ducts, damaging peribiliary glands and resulting in strictures.^2^ Nevertheless, there remains debate about the contribution of fibrin microthrombi to its occurrence.

While we have found peribiliary vascular fibrin in second to fourth order bile ducts of non-transplanted livers.^2^ Other authors examining the ends of ducts resected before biliary anastomosis report none, although a significant relation between arteriolonecrosis and PTC has been reported;^3^ it is possible that both are manifestations of the same phenomenon. The similarity of the cholangiographic appearance of PTC with that of hepatic arterial thrombosis has also suggested an ischemic origin, and prompted the descriptive term “ischemic-type biliary lesion.”

The origin of the presumed microthrombi has been questioned. The observation that fibrinolysis, associated with disseminated intravascular coagulation, is common in brain death suggests an origin after retrieval,^4^ while observations that many donors are pro-thrombotic are in favor of a donor origin.^5^ Observations that D-dimers, which are products of fibrin degradation, are washed out of livers during normothermic and hypothermic perfusion provide further evidence of the presence of fibrin,^6,7^ but not of its temporal origin. No prior heparin administration is permitted in DCD donors in the United Kingdom, a fact suggested to be associated with fibrin formation during donation. In contrast, in countries like Belgium, where pre-mortem heparin is permitted, cholangiopathy still occurs and in our previous work we have demonstrated D-dimers in livers from donation after brain death (DBD) donors who are fully heparinized at the time of retrieval.^6^

Whatever the temporal relation to retrieval, the presumed association of PTC with vascular thrombosis prompted researchers to explore fibrinolytic regimens to prevent PTC occurrence, such as by instillation of recombinant tissue plasminogen activator (TPA) in the hepatic artery during implantation.^8^ In spite of encouraging results in early pooled studies,^9^ excess bleeding and variable efficacy led some to discontinue the practice.^10^

One explanation for the varying efficacy of TPA protocols may be that they were administered without a source of plasminogen. TPA acts by converting plasminogen to plasmin, with plasmin being the principal fibrinolytic agent. TPA, such as alteplase (Actilyse, Boehringer Ingelheim, Germany) and a source of plasminogen, could be administered to a liver undergoing normothermic machine perfusion (NMP), ensuring optimal plasmin efficacy in a setting where bleeding is of no consequence. Encouraged by early experience where the liberation of D-dimers during NMP was much greater in the presence of alteplase, and using fresh frozen plasma (FFP) as a source of plasminogen,^11^ we decided to embark on a randomized trial to formally assess its efficacy in the prevention of PTC. Before that, we needed to assess the risk of post-implant bleeding and determine the most effective regimen.

Alteplase was selected on account of its non-immunogenic recombinant nature, its short half-life (3–5 min in vivo), and good efficacy. There are no data on the synthesis of plasminogen during NMP from previous studies,^12,13^ and although we presumed the liver would start producing plasminogen soon after starting NMP, we wanted adequate exogenous plasminogen to facilitate thrombolysis immediately NMP began. Purified plasminogen was not available, so FFP was chosen as the plasminogen source. An alternative strategy using plasmin itself was not possible since a clinical-grade product was not available.

FFP also contains alpha-2-antiplasmin,^14^ a potent inhibitor of plasmin with a rate constant of just one second.^15^ We did not know whether it would be optimal to infuse FFP and alteplase separately into the hepatic artery cannula to minimize the time available for mixing and subsequent deactivation of plasmin by antiplasmins, or whether they could be infused into the portal blood reservoir where mixing would take place and plasminogen activated sometime before it reached the artery, with the possibility that the plasmin would be deactivated before getting to where it was intended. In addition, we wanted to determine the optimal and safe dose of alteplase and FFP. To address these questions, we devised a pilot study looking at sequentially ascending doses of alteplase delivered into the arterial cannula, followed by evaluation of the infusion of the preferred alteplase dose into the portal blood reservoir; finally, we looked at increased doses of FFP. FFP would also contain complement, which might potentiate reperfusion injury in a way that NMP in the absence of complement avoids.

The primary endpoint of the study was post-implant blood loss, a necessary safety endpoint before embarking on a large-scale clinical trial. The main efficacy endpoint was the release of D-dimers into the perfusate, a reflection of fibrinolytic activity. The study objective was to define the optimal protocol to take forward to a definitive clinical trial of alteplase with FFP for the prevention of post-transplant cholangiopathy in livers undergoing NMP before transplantation.

## METHODS

### Trial design

This was a pilot, phase 1, pragmatic, sequential dose escalation, safety study. Donor livers were randomized into 1 of 5 groups, each comprising 8 DBD livers and 8 controlled (Maastricht 3 or 4) DCD livers. Four groups received alteplase with FFP, and the fifth group received FFP alone as controls (Table 1). Randomization was organized such that of the first 20 livers, 4 were randomized to control and 16 to receive protocol A; the next 20 were either randomized to control (n=4) or treatment B (n=16), and so on. Randomization was hoped to reduce bias in enrollment into the study and in cohort-by-time effects. It did run the risk of bias by operative learning, but the strict protocols for administration and blinding of the transplant surgeons to the results were hoped to mitigate such effects.

**TABLE 1:** Protocols used in the study

	Alteplase (tissue plasminogen activator, TPA)			Fresh frozen plasma (FFP)		
Protocol	Bolus (mg)	Infusion over 1 h (mg)	Delivery route	Bolus (mL)	Infusion over 1 h (mL)	Delivery route
A	2	8	Hepatic artery cannula	50	200	Hepatic artery cannula
B	4	16	Hepatic artery cannula	50	200	Hepatic artery cannula
C	2	8	Portal reservoir	50	200	Portal reservoir
D	2	8	Portal reservoir	0	0	500 mL in place of HAS in the prime solution
E	0	0	Not applicable	50	200	Portal reservoir

*Note:* 10 mg alteplase (recombinant tissue plasminogen activator, TPA) was reconstituted and made up to 50 mL with normal saline. One bag of fresh frozen plasma comprised a volume of ~250 mL. For protocol D, the 500 mL of 5% human albumin solution (HAS) used in the prime solution was replaced by 2 units (~500 mL) of fresh frozen plasma; there was no separate infusion of FFP.

The intention was to increase the dose of TPA incrementally, from 10 mg to 20 mg, to 50 mg, but interim analysis of D-dimer levels revealed no difference between 10 and 20 mg doses, so further alteplase dose increments were stopped, and the protocols were altered to explore portal delivery of alteplase and doubling the FFP dose (Table 1). No DCD donor received heparin before withdrawal of treatment.

The high prevalence of occult fibrin in DBD livers,^6^ as well as DCD livers, led us to include both donor types in the study, since the D-dimer load had been shown to be associated with graft survival and not just cholangiopathy. A group size of 16 was selected pragmatically to provide sufficient data on D-dimer release with TPA to inform a future study. Study groups D and E were to be finalized based on the analysis of bleeding complications and D-dimer concentrations from the preceding groups. In the end, group C received 10 mg alteplase with FFP infused into the portal reservoir, and group D replaced the human albumin solution (HAS) in the prime with FFP; we did not pursue a 2-hour duration alteplase infusion as originally envisaged.

The development of the trial was discussed with the National Institute for Health and Care Research Blood and Transplant Research Unit Patient and Public Research Panel, and was approved by the Cambridge East Research Ethics Committee (21/EE/0237). It was conducted in accordance with both the Declarations of Helsinki and Istanbul, and registered with the ISRCTN registry (ISRCTN15211703). The trial took place in 2 liver transplant units in the United Kingdom. All participants gave written, informed consent.

### Participants

Adults on the waiting list for a liver transplant were eligible to participate, provided they were not allergic to gentamicin, latex, or alteplase. Any DBD liver or DCD liver that had not previously undergone hypothermic oxygenated perfusion or in situ normothermic regional perfusion (NRP), was eligible for inclusion provided a decision had already been made for it to undergo NMP. The decision to subject a liver to NMP was made by the operating surgeon, independent of the study, and took into account logistic, donor, and recipient factors.

### Interventions

Livers were perfused using the *metra^®^* (OrganOx Ltd, Oxford, UK) device according to the manufacturer’s instructions. Either 500 mL Gelofusine (n=4) or 5% HAS (n=60) was used to suspend the red cells for perfusion for protocols A, B, C, and E, while 2 units of FFP were used for protocol D. FFP of blood group A or AB was used, depending on the liver blood group, to avoid anti-A or anti-B antibodies binding during perfusion. The perfusate was supplemented with 10000 units of heparin at the start, followed by infusion of 833 units/hour.

Alteplase was reconstituted and made up to a final volume of 50 mL in normal saline, and a loading dose of 20% delivered into the circuit at the start of liver perfusion, with the remainder infused over the next 60 minutes. FFP came in bags of median volume 267 mL (range 200–299 mL); 50 mL were given at the start of perfusion, and the remainder infused at a rate of 200 mL/hour. For protocols A and B, the alteplase and FFP infusions were delivered into separate lumens of a three-way tap on the hepatic artery cannula, and for group C, into a three-way tap on top of the portal reservoir, such that they mixed for the first time at that point. For protocol D, alteplase alone was infused into the portal reservoir, which already contained FFP. For protocol E, a bolus of 50 mL FFP was given and the rest of the unit infused over the next hour, without alteplase.

Perfusate and bile samples were taken at intervals to assess viability, and a decision to transplant the liver was made by the on-call surgeon, using standard viability criteria (Supplemental Table S1, http://links.lww.com/LVT/B85). Perfusate samples were immediately centrifuged, snap frozen in liquid nitrogen, and transferred to a freezer at −80 °C for later analysis. A 2-hour time point was chosen for D-dimer measurements based on previous experience with alteplase and FFP, suggesting that beyond this time, levels tended to plateau or fall^6^ (see also Supplemental Figure S1, http://links.lww.com/LVT/B85).

D-dimers were measured using latex immunoturbidimetry with the D-dimer HS assay on the ACL TOP 750CTS analyzer (Werfen, Barcelona, Spain) according to the manufacturer’s instructions. To compensate for different volumes of red cells and FFP in the perfusate, and following FFP infusions in groups A, B, C, and E, the D-dimer concentrations were multiplied by the estimated perfusate volume at 2 hours to get the total perfusate D-dimer load. The 2-hour perfusate volume was calculated based on the known volume of the prime and the hemoglobin concentrations before the liver was on board and at 2 hours, that is, [Hb]_prime_ × volume_prime_=[Hb]_2 hours_ × volume_2 hours_. Livers were weighed before cannulation, but following bench preparation, the weight-adjusted D-dimer release was reported.

The duration of alteplase activity once treatment had ceased was measured in a cohort from protocol D who had received 10 mg alteplase, using the Technozym tissue plasminogen activator (TPA) antigen ELISA (Technoclone, Vienna, Austria) performed in accordance with the manufacturer’s instructions. Plasminogen concentrations in FFP were measured using the chromogenic plasminogen assay on the ACL TOP 750CTS (Werfen, Barcelona, Spain), in accordance with the manufacturer’s instructions.

At the end of NMP, the livers were flushed with 2 L of ice-cold University of Wisconsin solution before implantation.

### Outcome measures

The primary endpoint was blood loss post-implantation of the liver, determined by suction volumes, including cell salvage and weighed swabs. To determine the efficacy of thrombolysis, the key secondary endpoints were the perfusate D-dimer concentrations after 2 hours of perfusion and the incidence of PTC. Other post-transplant outcomes are defined in Supplemental Table S2, http://links.lww.com/LVT/B85.

### Statistical considerations

The sample size was based on prior experience of D-dimer release in the belief that if there was a significant difference between treatment groups of 16 livers, it would manifest. No formal sample size calculation was undertaken.

The randomization sequence was determined by the senior author by generating a random sequence of numbers in Excel (Microsoft Corporation, USA), with separate sequences for DBD and DCD livers and blocks of 10 for each. Randomization for each treatment protocol and each donor liver type (DBD or DCD) was concealed in sealed envelopes; these were opened once a decision had been made to perfuse the liver.

Patients on the waiting list in each center were approached to consent. No formal blinding of the surgical team was undertaken, and on no occasion did they interfere with randomization or allocation. A record of the recipient’s involvement was made in their electronic patient record, but treatment allocation was not mentioned.

Treatment group data are summarized as median and interquartile ranges, and, where appropriate, between-group comparisons are made using the Kolmogorov–Smirnov test and multiple group comparisons using the Kruskal–Wallis test. Analyses used GraphPad Prism v10.3.0 for macOS (GraphPad Software, Boston, USA). Data are available upon reasonable request to the lead author.

## RESULTS

**FIGURE 1:**
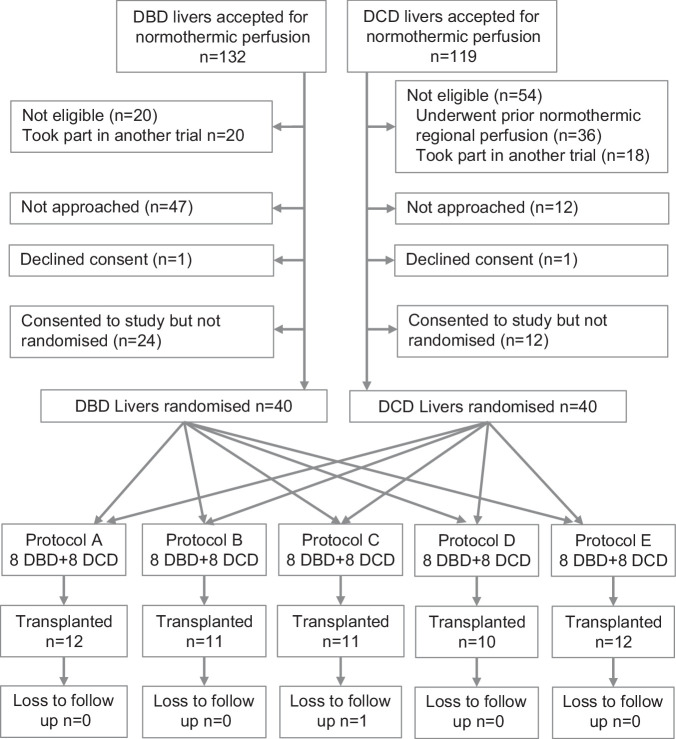
Consort diagram for recruitment.

Eighty livers were recruited between 15th April 2022 and 29th April 2024, resulting in 56 transplants (24 DCD, 32 DBD), one of which took place at a non-participating center after the liver had been declined for use at the participating center based on NMP parameters (Figure 1). There was a 1-year follow-up for all participants. The donor liver demographics are detailed in Table 2 by treatment group, and Supplemental Table S3, http://links.lww.com/LVT/B85, by donor type, and the post-transplant data are shown in Table 3. Of those livers not randomized, 38 were recruited to competing studies ongoing at the same time, and 36 underwent in situ NRP. Fifty-nine patients were not approached to take part, mostly because of the short time period between listing for transplant and admission; 2 declined to consent to the study, and 36 consented to take part but were not randomized either based on surgeon preference to either use, or not use, alteplase and FFP outside of the study, liver trauma, non-availability of alteplase, or non-availability of research staff. All participants received standard post-transplant care.

**TABLE 2:** Donor liver data

	A	B	C	D	E
Protocol	10 mg TPA +FFP into the HA cannula	20 mg TPA + FFP into the HA cannula	10 mg TPA + FFP into the portal reservoir	10 mg TPA in portal + 500 mL FFP in prime	Control: FFP infusion alone
Number of perfused livers	16	16	16	16	16
Donor age	47 (34–60)	58 (49–60)	49 (34–58)	56 (39–64)	46 (40–55)
Donor liver weight (kg)	1.7 (1.4–2.0)	1.7 (1.6–2.1)	1.8 (1.6–2.2)	1.7 (1.6–2.1)	1.7 (1.4–2.0)
UK DLI for DBD livers	0.9 (0.9–1.0)	1.0 (0.8–1.3)	0.9 (0.8–1.0)	1.0 (0.9–1.3)	1.1 (1.0–1.2)
UK DLI for DCD livers	2.3 (2.1–2.5)	2.0 (1.6–2.2)	2.0 (1.7–3.4)	2.1 (1.7–2.4)	1.8 (1.6–2.0)
US DRI for DBD livers	1.4 (1.3–1.9)	1.7 (1.6–2.1)	1.6 (1.4–2.2)	1.9 (1.4–2.5)	1.8 (1.5–2.1)
US DRI for DCD livers	2.7 (2.2–3.0)	2.9 (2.6–3.1)	2.4 (2.2–3.0)	2.5 (2.1–3.1)	2.3 (2.1–2.6)
ET-DRI for DBD livers	1.5 (1.4–1.7)	1.6 (1.5–2.0)	1.6 (1.4–2.1)	1.8 (1.5–2.3)	1.8 (1.6–2.0)
ET-DRI for DCD livers	2.7 (2.4–3.4)	2.8 (2.6–3.3)	2.6 (2.3–2.9)	2.57 (2.2–3.1)	2.39 (2.1–2.6)
Withdrawal to arrest (DCD livers) (min)	14 (11–17)	24 (12–39)	13 (9–24)	16 (9–48)	14 (9–30)
Arrest to in situ cold perfusion (DCD livers) (min)	12 (11–13)	13 (8–15)	13 (12–15)	13 (11–14)	14 (10–19)
Withdrawal to in situ cold perfusion (DCD livers) (min)	27 (22–39)	38 (26–48)	30 (22–34)	26 (21–61)	26 (21–48)
Duration of CIT before NMP (min)	398 (340–499)	424 (406–506)	464 (379–514)	464 (388–520)	451 (384–499)
Duration of NMP (min)	615 (465–738)	481 (350–717)	502 (363–703)	418 (347–549)	566 (435–699)

*Notes:* Each treatment protocol contains 8 DBD and 8 DCD livers. Values are quoted as median (interquartile range).

There was no liver weight recorded for one DCD liver on protocol D. Supplemental Table S3, http://links.lww.com/LVT/B85, quotes the calculated risk indices separately for DBD and DCD livers.

Abbreviations: CIT, cold ischemic time; ET-DRI, EuroTransplant donor risk index; FFP, fresh frozen plasma; HA, hepatic artery; NMP, normothermic machine perfusion; TPA, tissue plasminogen activator (alteplase); UK DLI, United Kingdom donor liver index; US DRI, United States donor risk index.

**TABLE 3:** Post-transplant data

	A	B	C	D	E
Treatment protocol	10 mg TPA + FFP into HA	20 mg TPA + FFP into HA	10 mg TPA + FFP into portal	10 mg TPA into the portal, 500 mL FFP in the prime	Control: FFP infusion alone
Number of perfused livers	16	16	16	16	16
Number of transplanted livers	12	11	11^a^	10	12
Macrosteatosis of transplanted livers	11× <5% 1×5%–33%	10× <5% 1×33%–66%	6× <5% 1×5%–33% 1× >66% 3× no biopsy	7× <5% 2×5%–33% 1× no biopsy	10× <5% 2×5%–33%
Recipient UKELD	53.4 (52.5–54.9)	56.0 (52.2–58.9)	55.3 (49.9–59.4)	55.3 (50.3–60.6)	51.8 (48.5–59.2)
Recipient MELD-Na	15.7 (14.9–15.2)	19.3 (14.3–22.3)	17.4 (12.5–22.2)	18.6 (11.4–24.3)	15.4 (12.1–23.8)
Retransplants	0	0	0	0	1
Superurgent (national priority) transplants	0	1	0	0	1
Number of units of PRC before reperfusion	3 (2–5)	3 (2–5)	2 (0–5)	2 (0–4)	6 (1–9)
Number of units of PRC after reperfusion	3 (0–4)	3 (0–6)	1 (0–3)	4 (1–5)	2 (1–5)
Blood loss pre-implant (mL)	2547 (1257–6245)	2240 (1369–5410)	1200^b^ (1025–2350)	3195 (1400–4107)	3502 (2119–7657)
Blood loss post-implant (mL)	3684 (1881–5433)	4006 (2250–6000)	1794^b^ (966–3900)	4398 (2061–6384)	3929 (1441–7550)
Total blood loss (mL)	7110 (3138–10750)	8133 (5364–11,200)	3319 (1695–6350)	7843 (3468–12,389)	7331 (4467–14,356)
Number returned to the theater within 24 h for bleeding	0	0	0	0	0
Number packed for bleeding	0	1	0	0	1
Incidence of post-reperfusion syndrome	0/12	4/11	0/10	0/10	0/12
MEAF score	2.4 (1.2–4.8)	3.6 (2.5–8.0)	5.1 (2.3–6.6)	3.6 (2.0–5.8)	3.3 (1.6–5.7)
EAD excluding PNF	0/12	4/10	3/10	3/9^c^	0/12
AKIN stage ≥2	4/12	3/10	2/10	2/9^c^	6/12
ICU stay (days)	3 (2–5)	3 (2–4)	3 (2–8)	3 (2–5)	5 (2–16)
Hospital stay (days)	25 (13–38)	15 (12–26)	14 (12–19)	18 (12–27)	31 (12–45)
Number undergoing MRCP	5/12	4/10	5/10	4/9^c^	8/12
Bile anastomotic leak	2/12	0/10	0/10	0/9^c^	0/12
Bile anastomotic stricture	1/12	0/10	1/10	0/9^c^	1/12
Post-transplant cholangiopathy					
Hilar stricture only	0/12	1/10 (RHAT)	0/10	1/9^c^	0/12
Intrahepatic strictures	0/12	1/10	1/10 (severe arterial stenosis)	1/9^c^ (RHAS)	1/12 (left lobe strictures and left HAT)
Incidence of common hepatic artery thrombosis	2/12	0	0	0	0
Graft and patient survivals					
Graft losses	2 HAT, days 15 and 16^d^	1 PNF day 4	0	1 PNF day 2	0
12-month graft survival (death censored)	83%	90%	100%	90%	100%
Deaths	1: Pulmonary hypertension day 245	None	1: CVA and multiorgan failure day 217	1: PNF day 2	1: COVID day 20
12-month patient survival	83%	100%	90%	90%	92%
12-month graft survival not censored for death	75%	90%	90%	90%	92%

*Note:* Data are median and interquartile ranges, or absolute numbers, unless otherwise stated.

^a^ One liver was declined for transplantation by the study centers and transplanted successfully at a third center, for which no post-transplant data beyond day 7 are available.

^b^ Accurate blood loss data before and after liver implantation were missing for 2 cases.

^c^ Denominator of 9 since one liver was lost early from PNF.

^d^ HAT on day 16 was secondary to complications arising from a liver biopsy.

Abbreviations: AKIN, Acute Kidney Injury Network; COVID, SARS-CoV-2 coronavirus infectious disease; CVA, cerebrovascular accident; EAD, early allograft dysfunction; FFP, fresh frozen plasma; HA, hepatic artery; HAT, hepatic artery thrombosis (entire arterial inflow thrombosed); HCC, hepatocellular carcinoma; ICU, intensive care unit; MEAF, Model for Early Allograft Function score; MELD-Na, Model for End-Stage Liver Disease—Sodium; MRCP, magnetic resonance cholangiopancreatography; PRC, packed red cells; PNF, primary nonfunction; RHAS, stenosed replaced right hepatic artery; RHAT, thrombosed accessory right hepatic artery; TPA, recombinant tissue plasminogen activator (alteplase); UKELD, United Kingdom Model for End-Stage Liver Disease.

### Bleeding

**FIGURE 2:**
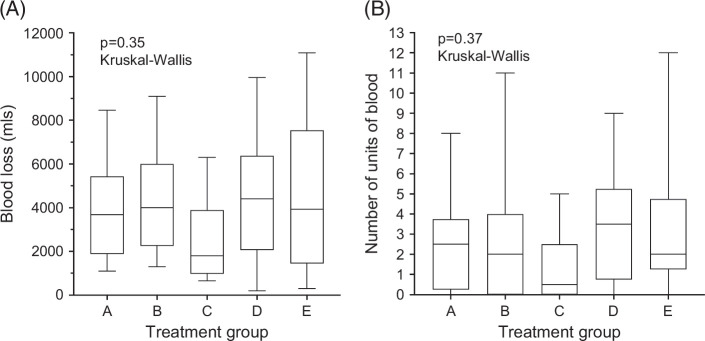
(A) Post-reperfusion blood loss and (B) blood transfusion requirement by treatment group. There is no significant difference between treatment groups and the control group in either recorded blood loss or blood units administered post-implantation.

There was no significant difference in the primary endpoint of post-reperfusion blood loss between groups, in particular, no suggestion of excess bleeding in the groups whose livers were treated with alteplase compared with the control group (Figure 2). Similarly, there was no significant difference in the requirement for transfusion of blood post-implant in any group compared with the control group. No patients were returned to surgery for bleeding. Excess intraoperative bleeding requiring perihepatic packing was necessary in 1 liver in the control group and 1 in group B. The degree of macrosteatosis, which was low in most livers, did not appear to affect post-implant blood loss.

### Treatment efficacy

**FIGURE 3:**
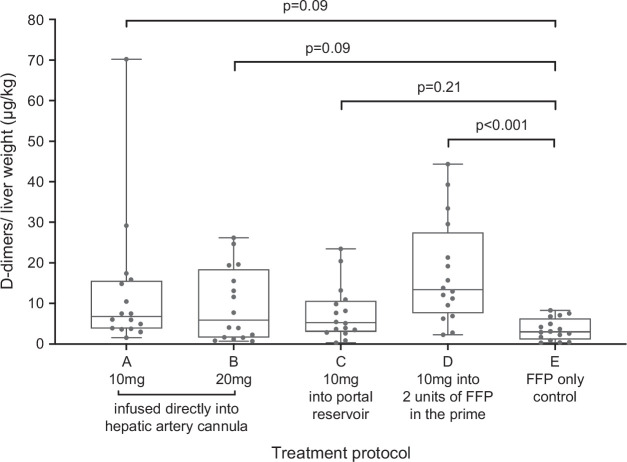
Total D-dimer content of perfusate at 2 hours adjusted for liver weight. To calculate the total D-dimer content, the D-dimer concentration was measured at 2 hours. The volume of the perfusate at 2 hours was calculated using the perfusate hemoglobin concentration before the liver was added, and at 2 hours, assuming the hemoglobin concentration would not have changed, and the volume of perfusate before the liver was added. Hence [Hb]_prime_ × volume_prime_=[Hb]_2 hours_ × volume_2 hours_. The total D-dimer release was then adjusted for the weight of the liver after bench preparation and immediately before cannulation. The weight was missing from 1 DCD liver in group D, so the median of the other DCD livers in group D was used for this liver. The graph shows that group D had the highest D-dimer release compared with the control group E (between-group statistical comparisons by Kolmogorov–Smirnov test). Abbreviations: DCD, donation after circulatory death; FFP, fresh frozen plasma.

Figure 3 shows that the treatment groups were associated with greater D-dimer release than the FFP alone control group E, significantly more in the case of group D, suggesting that D-dimer release was limited by a low concentration of plasminogen.

Six patients developed post-transplant cholangiopathy, 1 in the control group and 5 in the treatment groups (Supplemental Table S4, http://links.lww.com/LVT/B85). Two cases were associated with thrombosed hepatic arteries, and 2 with severe arterial stenoses requiring angioplasty, of which 1 had multiple intrahepatic arterial stenoses; this latter case had a very low 2-hour D-dimer load (0.32 µg/kg), suggesting that cholangiopathy was not related to peribiliary vascular thrombus. Both of the remaining cases had a limited disease distribution affecting part of the right lobe of the liver, raising the possibility of a missed replaced right hepatic artery branch in one, which could not be verified on computed tomographic angiography, while the other had a <50% stenosis of a replaced right hepatic artery; both released large amounts of D-dimers. The delayed time to diagnosis of one of these (day 155) also raises the possibility of recurrent primary sclerosing cholangitis in his DBD donor liver. In summary, the incidence of post-transplant cholangiopathy, ignoring those cases attributable to arterial complications, as recommended by the Innsbruck consensus,^1^ was 2 out of 43 (4.7%) of those treated with alteplase and FFP. The supplementary digital content provides perfusate chemistry for all the non-thrombotic cholangiopathy cases (Supplemental Figures S2–S8, http://links.lww.com/LVT/B85).

### Safety endpoints

Graft utilization was similar in all treatment groups, with between 10 and 12 out of the 16 perfused livers. Following transplantation, post-reperfusion syndrome only occurred in 4 (36%) patients in group B, an incidence of 7.1% overall.

Acute kidney injury occurred in between 20% and 40% of treatment group livers, and 50% of controls, a difference that was not significant with the numbers concerned. The model for early allograft function (MEAF) score was not significantly different between groups (*p*=0.68, Kruskal–Wallis). The study was not powered to show small differences between groups in these endpoints.

Two livers were lost to primary nonfunction (PNF), neither related to the treatment allocation: both are detailed in the supplementary digital content (Supplemental Figures S9-S11, http://links.lww.com/LVT/B85).

### Duration of alteplase activity

**FIGURE 4:**
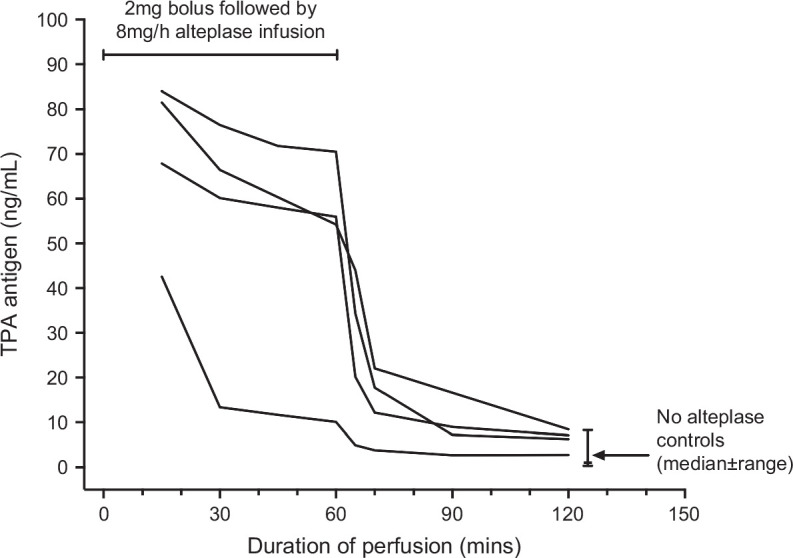
TPA antigen concentration during and after infusion of 10 mg TPA as per protocol D. The alteplase infusion finished 60 minutes after perfusion began. Abbreviation: TPA, tissue plasminogen activator.

TPA antigen was measured in 4 livers in protocol D receiving 10 mg alteplase. There was a rapid loss of TPA antigen after 60 minutes, with minimal levels 60 minutes later (Figure 4). TPA antigen measured in 5 livers that had not received alteplase revealed a low basal concentration (median 1.04 ng/mL, IQR 0.34–6.04).

### Plasminogen concentration in FFP and untreated perfusate

The plasminogen concentration of FFP was a median (IQR) 91 (85–95) U/dL in the 6 FFP units sampled. In contrast, plasminogen was undetectable (<10 U/dL) from time 0 through 120 minutes of perfusion in the perfusate of 5 livers that had undergone NMP without alteplase and FFP.

## DISCUSSION

This pilot study has demonstrated the feasibility and safety of combining alteplase with FFP in livers undergoing NMP, without increasing perioperative blood loss or transfusion requirements following transplantation. It has also demonstrated that the combination is associated with the release of more D-dimers into the perfusate than simple perfusion with FFP alone; that livers undergoing NMP produce negligible amounts of plasminogen in the first hour of perfusion so supplementation is necessary; that 2 units of FFP are better than 1 for release of D-dimers; and that 10 mg alteplase is as effective as 20 mg alteplase in prompting D-dimer release.

The study addressed several other issues. Concerns that combining alteplase with FFP in the portal reservoir would result in early neutralization of plasmin were unfounded. This is probably explained by the conversion of plasminogen to plasmin by alteplase being much more effective if the plasminogen is first bound to fibrin.^16^ This observation, confirmed by protocol C, allowed us to explore replacing HAS with FFP in the initial perfusate (“the prime”), in the knowledge that sufficient plasminogen was likely to remain until the alteplase infusion finished, 60 minutes into the perfusion. Delivering alteplase directly into the portal reservoir via the pre-existing three-way tap was technically more straightforward than delivery directly onto the arterial cannula, which required the lid of the liver bowl to be slightly ajar to allow passage of the infusion tubing. The rapid turnover of blood in the portal reservoir, with the portal flow being typically around 1.2 L/min in a circuit volume of 2 L, meant there was little delay in the alteplase reaching the hepatic artery and its branches. This arrangement was simpler to set up, since it did not require intervention in the operative field or liver bowl.

Although the study was small, and the fibrin loads within livers were relatively heterogeneous, we believe the study provided sufficient evidence to support the notion that 10 mg alteplase was as effective as 20 mg alteplase. While 20 mg was not associated with more blood loss, it costs 50% more than the 10 mg dose in the United Kingdom. Moreover, the fact that doubling the amount of FFP by using it in the prime solution in protocol D suggested that fibrinolysis was limited by plasminogen availability in the other protocols. This regimen will be used in a properly powered prospective study to look at the prevention of post-transplant cholangiopathy.

The occurrence of 6 cases of post-transplant cholangiopathy in this study is noteworthy, although the study was not powered to examine the effect of thrombolytic treatment on its incidence. Two cases were associated with arterial thrombosis, and 2 with severe arterial stenoses; these arterial complications are believed sufficient to explain the occurrence of the cholangiopathy in those cases. The remaining 2 cases were limited to the right-sided ducts, specifically the right anterior and posterior sectoral ducts in one case and the right posterior sectoral duct in the other. They were associated with high perfusate D-dimers, suggesting that these 2 cases may have been genuine post-transplant cholangiopathy and the treatment insufficient, although the limited disease distribution is also suspicious for a missed replaced right hepatic arteries in one case, and recurrence of primary sclerosing cholangitis in the other, particularly as the latter was in a DBD liver from a donor in their 40s with <9 hours cold ischemia and diagnosed 155 days post-transplant.

This study does have limitations. Ideally, a study of fibrinolysis would use pure plasmin or use purified plasminogen with alteplase to avoid any confounding problems with the other proteins present in FFP, such as alpha-2 antiplasmin and complement. It would allow a set dose of plasminogen to be delivered, avoiding the variability in using different bags of FFP. Pure plasminogen has been used with alteplase to clear peribiliary plexus thrombi in perfusions of discarded livers.^17^

At the start of the study, investigators believed 50 mg alteplase would provide optimal fibrin clearance similar to the recent experience from Mount Sinai,^18^ believing that fibrinolysis would be limited by the amount of circulating alteplase not, as it turns out, by the amount of circulating plasminogen. The study was originally developed around the premise that more alteplase would cause more fibrinolysis, and hence the plan for increasing doses of alteplase up to 50 mg. Fortunately, the structure of the study allowed us instead to evaluate increasing the amount of FFP.

The use of FFP alone in the prime solution merits comment, and in particular, whether it placed the livers more at risk of microthrombi formation. Given that the perfusate contained an infusion of heparin, we believe it is unlikely that new thrombus would form. In control group E, the washout of D-dimers during NMP is similar to that reported where HAS has been used in other studies, where alteplase and FFP were not given, after adjusting for a circulating volume of around 2 L. Finally, placing red cells in FFP is physiologically more appropriate than suspending them in HAS or Gelofusine. The only concern would be whether the presence of complement in FFP contributed to greater reperfusion injury during NMP, but we saw no evidence of this.

It was not possible to accurately measure changes in vascular resistance, which we anticipate would reduce with treatment. The OrganOx *metra^®^* does not measure portal pressure and does not directly measure arterial flow, but instead calculates it from the flows in the portal vein and vena cava, which it reports to the nearest 0.1 L/min, and excludes losses due to bleeding.

We acknowledge that post-transplant cholangiopathy can be caused by a number of factors, as outlined in the introduction. The high prevalence of D-dimers in DBD livers suggests that other factors, in addition to microthrombi, are at play, such as warm ischemia and reperfusion injury. We believe the initial injury to the duct epithelium will not progress to cholangiopathy, provided there is a nest of cells within adjacent peribiliary glands within viable stroma capable of repopulating the damaged epithelium; it is the ischemic injury to these glands that promotes scarring rather than healing.

This study was envisaged as a prelude to a large, multicenter study looking at the efficacy of alteplase and FFP in the prevention of post-transplant cholangiopathy, a study that will require several hundred livers. Before embarking on such a study, it was important to ensure the optimal treatment regimen is employed, and this was the rationale for this study. Preclinical models, such as porcine perfusions, were not considered appropriate due to the differences in coagulation parameters and proteins. We acknowledge that a larger sample size per group would increase confidence in the data, and were heartened to read the experience from Mount Sinai, involving 56 DCD livers treated with 50 mg alteplase and FFP and with a median MELD of 20, higher than in our study, in which there was no increase in blood product usage.^18^

The ability of alteplase and FFP to clear fibrin, as demonstrated here, supports this combination treatment in conjunction with NMP as a possible treatment of choice for the prevention of cholangiopathy. While we and others have shown that hypothermic perfusion can flush some D-dimers from the vascular space,^6^ this is less efficient, as suggested by the observation that D-dimers continue to be shed after many hours,^7^ suggesting brief periods of hypothermic perfusion, as widely practiced, leave much fibrin remaining with a risk of stricture formation.

In conclusion, this randomized controlled pilot study has demonstrated that the ex situ fibrinolytic treatment of livers during NMP is not associated with increased blood loss once the liver has been implanted, and has provided more evidence that occult fibrin is present within donor livers. It has been shown that a combination of alteplase and FFP can enhance its clearance, that 10 mg alteplase is adequate, and that 2 units of FFP are better than 1 when combined with 10 mg alteplase.

## Supplementary Material
