# Supplementary material for: A pilot, two-center, sequential dose escalation safety study of alteplase with fresh frozen plasma during normothermic liver perfusion
Source: Liver Transpl. 2026 Feb 24;32(7):978–90. doi: 10.1097/LVT.0000000000000814 (PMC13275069; doi:10.1097/LVT.0000000000000814)
Supplement: Supplementary file 1 [file lvt-32-978-s001.docx]

**A pilot, two-centre, parallel group, ascending dose randomised controlled trial of alteplase with fresh frozen plasma during normothermic liver perfusion: Supplemental Digital Content**

# Table of contents

[Table of contents 1](#_Toc213086288)

[SDC Table 1. Cambridge viability criteria 2](#_Toc213086289)

[SDC Figure 1. D-dimer levels by duration of perfusion 3](#_Toc213086290)

[SDC Table 2. Definitions of recipient variables and post-transplant outcomes 4](#_Toc213086291)

[SDC Table 3. Donor liver data, separating DBD and DCD livers per treatment group 5](#_Toc213086292)

[SDC Table 4. Post transplant cholangiopathy cases 7](#_Toc213086293)

[Details of the subjects with post-transplant cholangiopathy 9](#_Toc213086294)

[Subject 1, treatment group B 9](#_Toc213086295)

[Subject 2, treatment group D 11](#_Toc213086296)

[Subject 3, treatment group B 13](#_Toc213086297)

[Subject 4, treatment group B 15](#_Toc213086298)

[Subject 5, treatment group D 17](#_Toc213086299)

[Subject 6, treatment group E 19](#_Toc213086300)

[Details of subjects suffering primary non function 21](#_Toc213086301)

[Subject in group B 21](#_Toc213086302)

[Subject in group D 23](#_Toc213086303)

[SDC References 26](#_Toc213086304)

# SDC Table 1. Cambridge viability criteria

|  | **PERFUSATE** | | | | **BILE*** | |
| --- | --- | --- | --- | --- | --- | --- |
|  | **ALT at 2 hours** (iu/L) | **Lactate**  mmol/L | **Glucose** mg/dL (mmol/L) | **pH** | **Glucose** mg/dL (mmol/L) | **pH** |
| **STANDARD RISK** | <6000iu/L | <2.8 mmol/L  @ 2 hours | >180mg/dL (10mmol/L) at 15min  *and*  falling after 2h | Maintained at 7.3 to 7.5 without bicarbonate supplementation beyond 2hours | <40mg/dL (2.2mmol/L) or ≥180mg/dL (10mmol/L) less than perfusate | >7.6  (>0.2 more than perfusate) |
| **HIGHER RISK** | 6000 to 10000 iu/L | 2.8 to 4 mmol/L @ 6 hours | <180mg/dL (10mmol/L)  @ 15 min *and* falling after a bolus of 25mls 20% dextrose | ≤20 ml 8.4% NaHCO_3_ between 2h and 4h | <80mg/dL (4mmol/L) or >90mg/dL (5mmol/L) less than perfusate | 7.5 to 7.6  (<0.2 more than perfusate) |
| **DO NOT USE** | >10000 iu/L | >4 mmol/L @ 6 hours | Not falling by 6h | Continued requirement for bicarbonate | No difference between perfusate and bile | No difference between perfusate and bile |

*Bile sampling should be simultaneous with perfusate sampling. Sampling a collection of bile formed over the previous hour will not be representative of the same time point as the perfusate and caution should be used in interpreting adverse readings in such cases.

Based on Predicting Early Allograft Function After Normothermic Machine Perfusion. *Transplantation*. Dec 1 2022;106(12):2391-2398. doi:10.1097/TP.0000000000004263

# SDC Figure 1. D-dimer levels by duration of perfusion

18 livers treated with alteplase during perfusion, and 6 untreated had perfusate sampled at intervals. The D-dimer concentration in perfusate plateaued aby 90 minutes and remained at similar level through to 180 minutes. For the purposes of this and other studies we selected 120 minutes as the point at which D-dimer concentration would be recorded. The lines within the shaded areas represent the median values and the shaded area the interquartile range.

# SDC Table 2. Definitions of recipient variables and post-transplant outcomes

Post transplant outcomes were defined as follows:

| **Outcome** | **Definition derivation** |
| --- | --- |
| AKI | Acute kidney injury was defined as a creatinine more than two-fold higher in the first 7 days than the value immediately pre-transplant, or the use of renal replacement therapy in the post-transplant period in a patient not previously requiring it (Acute Kidney Injury Network stage ≥2) ^1^. |
| EAD | Early allograft function after Olthoff *et al* ^2^ |
| ET-DRI | EuroTransplant donor risk index after Braat *et al* ^3^ |
| MEAF | Model for early allograft function calculated according to the method of Pareja *et al* ^4^. |
| MELDNa | Model for end-stage liver disease sodium after Kim *et al* ^5^ |
| PNF | Primary non function was defined as a failure of a liver to provide life sustaining function in the first 7 days post-transplant excluding failures due to hepatic artery thrombosis. |
| PRS | Post reperfusion syndrome was defined as a fall in mean blood pressure in the 5 minutes post reperfusion of >30% of that in the 5 minutes before reperfusion. |
| PTC | Post transplant cholangiopathy was defined according to the Innsbruck consensus meeting ^6^. |
| UK DLI | United Kingdom Donor Liver Index after Collett *et al* ^7^ |
| UKELD | United Kingdom model for End stage Liver Disease after Barber *et al*^8^ |
| US DRI | United States Donor Risk Index after Feng *et al* ^9^ |

# SDC Table 3. Donor liver data, separating DBD and DCD livers per treatment group

| **Donor type** | **DCD donors** | | | | | **DBD donors** | | | | |
| --- | --- | --- | --- | --- | --- | --- | --- | --- | --- | --- |
| **Protocol** | **A** | **B** | **C** | **D** | **E** | **A** | **B** | **C** | **D** | **E** |
|  | 10mg TPA +FFP into HA | 20mg TPA + FFP into HA | 10mg TPA + FFP into portal | 10mg TPA in portal + 500ml FFP in prime | Control: FFP infusion alone | 10mg TPA +FFP into HA | 20mg TPA + FFP into HA | 10mg TPA + FFP into portal | 10mg TPA in portal + 500ml FFP in prime | Control: FFP infusion alone |
| Number of perfused livers | 8 | 8 | 8 | 8 | 8 | 8 | 8 | 8 | 8 | 8 |
| Donor age | 60  (46-62) | 58  (57-61) | 51  (40-58) | 56  (27-61) | 43  (39-47) | 34  (30-47) | 53  (35-59) | 46  (20-64) | 57  (39-70) | 52  (46-63) |
| Donor liver weight (kg) | 1.8  (1.5-2.2) | 1.7  (1.5-2.0) | 1.9  (1.7-2.2) | 1.6  (1.5-2.1) | 2.0  (1.4-2.3) | 1.6  (1.3-1.8) | 1.9  (1.6-2.3) | 1.7  (1.5-2.2) | 1.7  (1.5-2.2) | 1.6  (1.3-1.9) |
| UK DLI | 2.3  (2.1-2.5) | 2.0  (1.6-2.2) | 2.0  (1.7-3.4) | 2.1  (1.7-2.4) | 1.8  (1.6-2.0) | 0.9  (0.9-1.0) | 1.0  (0.8-1.3) | 0.9  (0.8-1.0) | 1.0  (0.9-1.3) | 1.1  (1.0-1.2) |
| US DRI | 2.7  (2.2-3.0) | 2.9  (2.6-3.1) | 2.4  (2.2-3.0) | 2.5  (2.1-3.1) | 2.3  (2.1-2.6) | 1.4  (1.3-1.9) | 1.7  (1.6-2.1) | 1.6  (1.4-2.2) | 1.9  (1.4-2.5) | 1.8  (1.5-2.1) |
| ET-DRI | 2.7  (2.4-3.4) | 2.8  (2.6-3.3) | 2.6  (2.3-2.9) | 2.57  (2.2-3.1) | 2.39  (2.1-2.6) | 1.5  (1.4-1.7) | 1.6  (1.5-2.0) | 1.6  (1.4-2.1) | 1.8  (1.5-2.3) | 1.8  (1.6-2.0) |
| Withdrawal to arrest (mins) | 14  (11-17) | 24  (12-39) | 13  (9-24) | 16  (9-48) | 14  (9-30) |  |  |  |  |  |
| Arrest to *in situ* cold perfusion (mins) | 12  (11-13) | 13  (8-15) | 13  (12-15) | 13  (11-14) | 14  (10-19) |  |  |  |  |  |
| Withdrawal to *in situ* cold perfusion (mins) | 27  (22-39) | 38  (26-48) | 30  (22-34) | 26  (21-61) | 26  (21-48) |  |  |  |  |  |
| Duration of CIT before NMP (mins) | 363  (328-415) | 416  (398-425) | 476  (379-508) | 461  (388-514) | 422  (361-457) | 496  (392-569) | 506  (424-697) | 459  (379-552) | 464  (394-525) | 499  (438-562) |
| Duration of NMP (mins) | 587  (422-648) | 580  (374-742) | 424  (328-589) | 416  (320-645) | 514  (447-607) | 672  (465-872) | 383  (280-684) | 703  (502-781) | 447  (361-549) | 645  (412-726) |

Values are quoted as median (interquartile range). There was no liver weight recorded for one DCD liver on protocol D

CIT: cold ischaemic time;
ET-DRI: EuroTransplant donor risk index.
FFP: fresh frozen plasma ;
HA: hepatic artery;
NMP: normothermic machine perfusion;
TPA: tissue plasminogen activator (alteplase);
UK DLI: United Kingdom donor liver index;
US DRI: United States donor risk index;

# SDC Table 4. Post-transplant cholangiopathy cases

| **Subject** | **Protocol** | **Age DBD / DCD** | **Recipient liver disease** | **Hepatic artery** | **Post op day of diagnosis** | **2-hour D-dimers (µg/kg)** | **12 month bilirubin (µmol/L)**  **(NR:<20)** | **12 month ALP (IU/L)**  **(NR:30-130)** | **Affected ducts** |
| --- | --- | --- | --- | --- | --- | --- | --- | --- | --- |
| 1 | B | 58yo DCD | HCC and HCV | Normal | 91 | 19.4 | 7 | 260 | Cholangiopathy involving the right anterior and posterior sectoral ducts |
| 2 | D | 49yo DBD | PSC | Right hepatic artery stenosis <50% – not angioplastied | 155 | 44.4 | 10 | 129 | Isolated right anterior sectoral duct stricture |
| **Strictures secondary to significant arterial compromise** | | | | |  |  |  |  |  |
| 3 | B | 20yo DBD | ArLD | Thrombosed accessory right hepatic artery | 102 | 4.1 | 10 | 455 | Central biliary strictures involving the 1st order and 2nd order ducts. |
| 4 | C | 32yo DCD | HCC and HBV | Severe intrahepatic artery stenoses – failed angioplasty  (see SDC figure 4) | 76 | 0.3 | 18 | 179 | Stricturing of first order ducts at the hilum and in the periphery of both lobes |
| 5 | D | 19yo DCD | ArLD | Hepatic artery stenosis – successful angioplasty | 104 | 13.8 | 21 | 128 | Stricturing of first order ducts at the hilum |
| 6 | E | 47yo DCD | HCC and ADPKD | Thrombosed left hepatic artery; right hepatic vein stenosis requiring angioplasty, portal vein stenosis requiring stenting | 64 | 1.7 | 190 | 1581 | Strictures affecting confluence and left sided ducts only. |

ADPKD: autosomal dominant polycystic kidney (and liver) disease;
ALP: alkaline phosphatase;
ArLD: alcohol related liver disease;
HBV: Hepatitis B associated cirrhosis;
HCC: hepatocellular cancer;
HCV: Hepatitis C associated cirrhosis;
NR: Normal range.
PSC: primary sclerosing cholangitis;
yo: years old
Subject 1 had a withdrawal to arrest period of 111 minutes, an asystolic time of 9 minutes, and 404 minutes of cold ischaemia (CIT).
Subject 2 had a CIT of 522 minutes.
Subject 3 had a CIT 453 minutes.
Subject 4 had a withdrawal time of 13 minutes, asystolic period of 13 minutes, and CIT of 371 minutes;
Subject 5 had a withdrawal time of 13 minutes, asystolic period of 10 minutes, and 515 minutes of cold ischaemia;
Subject 6 had a withdrawal time of 9 minutes, asystolic period of 14 minutes, and 379 minutes of cold ischaemia.

# Details of the subjects with post-transplant cholangiopathy

## Subject 1, treatment group B

Subject 1 in SDC table 4 received a liver from a 58-year-old DCD donor with hypoxic brain injury who was 6 days in ITU. Timings were:

Withdrawal to arrest period of 111 minutes

Asystolic period of 9 minutes;

Extraction time: 29 minutes;

Cold ischaemia: 404 minutes;

NMP duration: 530 minutes

NMP perfusion chemistry is detailed in the figures below. The bile had a high pH and low glucose. The perfusate glucose was also low and the liver received several boluses of dextrose during the course of perfusion. Bile glucose was always lower than perfusate, between 1.0 and 1.9mmol/L (18 to 34mg/dL) (SDC Figure 2B).

The subject developed strictures in the right anterior and posterior sectoral ducts. CT revealed only a “mild stenosis” of the hepatic artery anastomosis.

**SDC Figure 2A Bile and perfusate pH during NMP for subject 1.**

**SDC Figure 2B Bile and perfusate glucose and perfusate lactate for subject 1**

## Subject 2, treatment group D

Subject 2 in SDC table 4 received a DBD liver from a 49-year-old donor on ITU for 3 days who died from meningitis.

Timings were:

Extraction time: 44 minutes;
Cold ischaemia: 522 minutes;
NMP duration: 492 minutes

The bile was not draining so the duct was recannulated and allowed to drain away from the standard receptacle, such that it was not possible to record bile chemistry. Perfusate chemistry is given in SDC figures 3A and 3B.

The liver developed a stenosis in a replaced right hepatic artery which had arisen from the donor’s SMA and was reconstructed onto the splenic artery stump. The stenosis was less than 50% the diameter of the artery and was felt not to warrant angioplasty. It subsequently developed stricture of the right anterior sectoral duct identified at 155 days. The recipient was transplanted for primary sclerosing cholangitis, raising the possibility that the stricture may have been recurrence of that disease.

**SDC Figure 3A. Perfusate glucose and lactate.**

**SDC Figure 3B. Perfusate pH**

## Subject 3, treatment group B

Subject 3 in SDC table 4 received a DBD liver from a 20-year-old donor on ITU for 4 days who died from an intracranial haemorrhage.

Timings were:

Extraction time 43minutes;

Cold ischaemia: 453 minutes;
NMP duration 839 minutes.

The liver had an accessory right hepatic artery which was anastomosed to the splenic artery stump. The liver had a persistently high glucose and lactate for 6 hours before finally falling, and perfusion was extended to ensure parameters fell to normal. The young donor age and low ALT (1243iu/L at 2 hours) explained the willingness to wait so long. Bile volume was low but chemistry was satisfactory.

Post transplant the reconstructed RHA was not visible on imaging, and the liver developed central strictures extending to second order ducts.

**SDC Figure 4A. Bile and perfusate glucose and perfusate lactate for subject 3 in SDC table 4
**

**SDC Figure 4B. Bile and perfusate pH for subject 3 in SDC table 4**

## Subject 4, treatment group B

Subject 4 in SDC table 4 received a DCD liver from a 32-year-old donor on ITU for 2 days who died from an intracranial haemorrhage.

Timings were:

Withdrawal to arrest period of 13 minutes;

Asystolic period of 13 minutes;
Extraction time 47minutes.

Cold ischaemia: 371 minutes;
NMP duration 485 minutes.

The perfusion was uneventful with a rapid fall glucose and a slightly delayed fall in lactate, with a 2-hour ALT 2423iu/L. The bile had an appropriately high pH and the glucose an appropriate low concentration compared to perfusate, reaching 1.0 mmol/L (18mg/dL).

**SDC Figure 5A. Bile and perfusate glucose and perfusate lactate for subject 4 in SDC table 4**

**SDC Figure 5B. Bile and perfusate pH for subject 4 in SDC table 4**

Angiography showed tight stenoses in 3 hepatic artery main branches within the liver parenchyma (SDC figure 5) which were believed to account for the appearance of post-transplant cholangiopathy.

**SDC Figure 6. Angiogram of the liver in subject 3 in SDC Table 3 showing three tight intrahepatic arterial stenoses.**


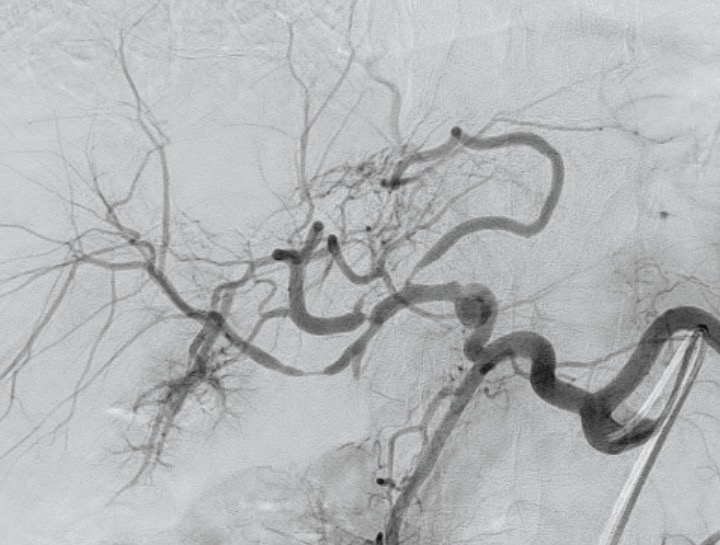


## Subject 5, treatment group D

Subject 5 in SDC Table 4 received a DCD liver from a teenage donor on ITU for 8 days who died from a hypoxic brain injury post cardiac arrest.

Timings were:

Withdrawal to arrest period of 13 minutes;
Asystolic period of 10 minutes;
35minutes extraction time;
Cold ischaemia: 515 minutes;
NMP duration 1181 minutes

The bile was slow to collect but had an appropriately high pH and the glucose an appropriately low concentration compared to perfusate, reading <1.0 mmol/L (18mg/dL) from the outset (SDC Figures 7A and 7B). He had a 2cm long hepatic artery stenosis that was successfully angioplastied, but developed a hilar stricture.

**SDC Figure 7A. Glucose and lactate for subject 5.**

**SDC Figure 7B. Bile and perfusate pH for subject 5**

## Subject 6, treatment group E

Subject 6 in SDC table 4 received a DCD liver from a 47-year-old donor on ITU for 6 days who died from a hypoxic brain injury.

Timings were:

Withdrawal to arrest period of 9 minutes;
asystolic period of 14 minutes;
45 minutes extraction time;
Cold ischaemia: 379 minutes;
NMP duration 533 minutes

The bile had an appropriately high pH and the glucose an appropriate low concentration compared to perfusate (SDC Figures 8A and 8B).

There were many vascular issues with this liver, including a left hepatic artery thrombosis, severe stenosis (3mm) of the extrahepatic portal vein and a right hepatic vein stenosis. There was consequent development in intrahepatic cholangiopathy affecting the left sided ducts, extending to the left first and second order ducts.

**SDC Figure 8A. Perfusate lactate and glucose and bile glucose**

**SDC Figure 8B. Bile and perfusate pH.**

# Details of subjects suffering primary non function

## Subject in group B

57-year-old DBD donor who died of an intracranial haemorrhage. 30 years previously he had been stabbed in the lower right chest and at retrieval it was noted that part of segments 8 and 4 of the liver had herniated through the diaphragm, with atrophy of the medial aspect of segment 4. The significance of this dysmorphism was not appreciated when the liver was placed on the *metra* in a manner that impaired venous drainage, with post-perfusion, pre-implant biopsy showing extensive intraparenchymal haemorrhage and confluent areas of severe hepatocyte loss in a perivenular distribution which was not present in the pre-perfusion biopsies.

Due to the extensive previous surgical history of the recipient, it had been elected to put the liver on NMP, and perfusion began at the same time as the patient was anaesthetised. The lactate was slow to fall which raised some concerns, but other parameters were satisfactory

Following implantation the liver did not work.

CIT 421 minutes; NMP duration 354 minutes

ALT 3562 at 1 hour, 3484 at 2 hours, 3882 at 4 hours.

**SDC Figure 9A : Perfusate and bile glucose and lactate during NMP.**

**SDC Figure 9B: Perfusate and bile pH during NMP.**

## Subject in group D

71-year-old DBD donor dying from an intracranial haemorrhage secondary to glioblastoma, first diagnosed on the admission CT.

371 mins CIT

ALTs 1016 at 1 hour, 1243 at 2 hours, 1452 at 4 hours. The liver behaved well during NMP with satisfactory parameters (SDC figures 10A and B)

**SDC Figure 10A Perfusate and bile glucose and lactate**

**SDC Figure 10B: Perfusate and bile pH during NMP.**

The recipient had undergone a liver resection previously. The donor liver was implanted and reperfused on the portal vein. There was a 99 minute delay in reperfusing the hepatic artery while haemostasis was gained. In the interval between portal and arterial reperfusion the central pressure increased (see SDC figure 10), opposing the portal perfusion pressure. Biopsies of the liver post NMP and before implantation were normal, but post reperfusion in the recipient prior to closure of the abdomen the biopsy showed extensive necrosis. Primary non function was attributed to warm ischaemia secondary to the high central venous pressure opposing forward portal venous flow for the duration it took to perform the arterial anastomosis.

**SDC Figure 11. Central venous pressure before and after reperfusion of the liver in the recipient, showing sustained venous hypertension .**

# SDC References

1. Lopes JA, Jorge S. The RIFLE and AKIN classifications for acute kidney injury: a critical and comprehensive review. *Clin Kidney J*. Feb 2013;6(1):8-14. doi:10.1093/ckj/sfs160

2. Olthoff KM, Kulik L, Samstein B, et al. Validation of a current definition of early allograft dysfunction in liver transplant recipients and analysis of risk factors. *Liver Transpl*. Aug 2010;16(8):943-9. doi:10.1002/lt.22091

3. Braat AE, Blok JJ, Putter H, et al. The Eurotransplant donor risk index in liver transplantation: ET-DRI. *Am J Transplant*. Oct 2012;12(10):2789-96. doi:10.1111/j.1600-6143.2012.04195.x

4. Pareja E, Cortes M, Hervas D, et al. A score model for the continuous grading of early allograft dysfunction severity. *Liver Transpl*. Jan 2015;21(1):38-46. doi:10.1002/lt.23990

5. Kim WR, Biggins SW, Kremers WK, et al. Hyponatremia and mortality among patients on the liver-transplant waiting list. *N Engl J Med*. Sep 4 2008;359(10):1018-26. doi:10.1056/NEJMoa0801209

6. Esser H, de Jong IEM, Roos FM, et al. Consensus classification of biliary complications after liver transplantation: guidelines from the BileducTx meeting. *Br J Surg*. Apr 30 2025;112(5):znae321. doi:10.1093/bjs/znae321

7. Collett D, Friend PJ, Watson CJ. Factors Associated With Short- and Long-term Liver Graft Survival in the United Kingdom: Development of a UK Donor Liver Index. *Transplantation*. Apr 2017;101(4):786-792. doi:10.1097/TP.0000000000001576

8. Barber K, Madden S, Allen J, et al. Elective liver transplant list mortality: development of a United Kingdom end-stage liver disease score. *Transplantation*. Aug 27 2011;92(4):469-76. doi:10.1097/TP.0b013e318225db4d

9. Feng S, Goodrich NP, Bragg-Gresham JL, et al. Characteristics Associated with Liver Graft Failure: The Concept of a Donor Risk Index. *Am J Transplant*. 2006;6:783-90.
